# CD44 Expression in Clear Cell Renal Cell Carcinoma (ccRCC) Correlates with Tumor Grade and Patient Survival and Is Affected by Gene Methylation

**DOI:** 10.3390/genes15050537

**Published:** 2024-04-24

**Authors:** Anastasios D. Papanastasiou, Stavros Peroukidis, Chaido Sirinian, Elisavet Arkoumani, Dimitrios Chaniotis, Adamantia Zizi-Sermpetzoglou

**Affiliations:** 1 Department of Biomedical Sciences, University of West Attica, 12243 Athens, Greece; 2 Panarkadikon General Hospital, 22100 Tripolis, Greece; 3 Molecular Oncology Laboratory, Division of Oncology, Department of Medicine, University of Patras, 26504 Patras, Greece; 4 Pathology Department, Tzaneion General Hospital of Piraeus, 18536 Piraeus, Greece

**Keywords:** CD44, renal cell carcinoma, immunohistochemistry

## Abstract

Clear cell RCC (ccRCC) represents the most common type of kidney cancer, with surgery being the only potential curative treatment. Almost one-third of ccRCC patients relapse either locally or as cases of distant metastases. Several biomarkers have been employed in order to separate ccRCC patients with better prognosis or to predict treatment outcomes, with limited results. CD44 is a membrane glycoprotein with multiple roles in normal development but also cancer. Recently, the CD44 standard isoform has been implicated in tumor progression and the metastasis cascade through microenvironment interactions. Here, through CD44 immunohistochemical staining of ccRCC patient samples and TCGA data analysis, we sought to elucidate the expression patterns (mRNA and protein) of CD44 in clear cell RCC and correlate its expression with clinicopathological parameters. We were able to show that CD44 expression presents a positive association with tumor grade and overall survival, predicting a worse patient outcome in ccRCC. In addition, our data indicate that the *CD44* mRNA upregulation can be attributed to reduced gene methylation, implicating epigenetic gene regulation in ccRCC development and progression.

## 1. Introduction

Renal cell carcinoma (RCC) accounts for 3% of all solid malignancies and is amongst the most lethal of urological cancers [1]. Clear cell RCC (ccRCC) represents the most common type of RCC, comprising approximately 60% of all renal tumors [2]. To date, the only potential curative treatment is surgery. However, 20–30% of patients with ccRCC develop local or distal recurrence within years of a nephrectomy [3]. The recurrence of ccRCC after surgery is a major factor that negatively affects patient survival. On the other hand, multiple unfavorable prognostic factors such as perineal fat invasion, tumor size as a continuous variable, size of the largest involved lymph node, and extra-nodal extension are neither reliable nor accurate enough for predicting the therapeutic response [4,5,6]. Recent studies have shown that a number of molecular and genetic biomarkers could play a role in predicting response to therapy [4,6,7]. 

CD44 glycoproteins are potentially important markers of tumor progression that participate in the metastatic cascade [8,9]. At least 20 different isoforms of the human CD44 lymphocyte-homing receptor/hyaluronase receptor have been described that arise from the differential splicing of up to 10 alternative exons, encoding the membrane-proximal extracellular protein domain [9,10].

The main receptor for hyaluronate acid is the CD44s (standard) [11]. CD44s expression was found in many tumor types including colorectal carcinoma [12], breast carcinoma [10], squamous carcinoma [13,14], and malignant melanoma [15]. Furthermore, expression of the CD44s was reported to positively correlate with increased survival in bladder cancer, fibrosarcoma, and gastrointestinal stromal tumors [16,17]. On the other hand, elevated expression of CD44s was associated with poor survival outcomes in pharyngeal and laryngeal cancer patients [14]. The expression patterns of CD44 in clear cell RCC remain unclear.

The aim of this study was to investigate the biological significance of CD44s expression in ccRCC. In addition, we were able to determine its association at the mRNA level with clinicopathological features and long-term outcome. Further, we provide preliminary evidence for a methylation-dependent upregulation of the *CD44* gene in clear cell RCC patient samples.

## 2. Materials and Methods

### 2.1. Tissue Specimens

Formalin-fixed paraffin-embedded postsurgical specimens from human ccRCC tissue were retrieved from the archives of Tzaneio General Hospital, Piraeus, Greece. The cohort included a total of 89 unrelated patients, who were diagnosed with clear cell RCC, and underwent a radical or partial nephrectomy. Histological evaluation and staging were based on the Fuhrman grading system and the TNM classification of the 2009 American Joint Committee on Cancer (AJCC), TNM staging system (7th edition). This study was conducted according to the principles laid out by the Declaration of Helsinki.

### 2.2. Immunohistochemistry and Immunohistochemical Evaluation

Representative 4 μm serial tissue sections were de-paraffinized in xylene and rehydrated in graded ethanol. Antigen retrieval and inhibition of endogenous peroxidase activity was performed by microwaving the slides in pH 6 citrate buffer and by treatment with 1% hydrogen peroxide, respectively, followed by a 1 h incubation with blocking solution. The tissue sections were subsequently incubated with anti-CD44 (Novocastra, clone F10-44-2) primary antibody (1:350). Immunohistochemical detection and visualization were performed according to manufacturers protocol (Envision detection kit, DAKO, Hamburg, Germany). 

**Figure 1:**
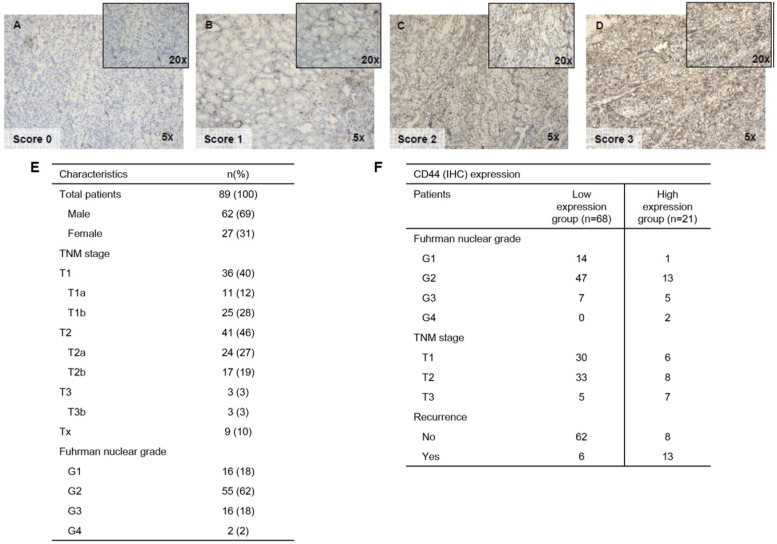
CD44 IHC stain of representative ccRCC cases depicting the membranous pattern of the CD44 protein and the relative scores employed in this study for assessing total protein expression in each case; (**A**) score 0, (**B**) score 1, (**C**) score 2, (**D**) score 3. Clinicopathological characteristics of the 89 ccRCC patients employed in this study (**E**). Clinicopathological characteristics of the CD44 low- and high-expression groups of patients with ccRCC (**F**).

All slides were assessed by two pathologists (A.D.P. and A.Z.S.) independently and blinded to the case. For the immunohistochemical evaluation of CD44, a semi-quantitative method was employed, taking into account the intensity of staining and the percentage of cells stained. The percentage of CD44 positive stained cell was scored as 1–25%, 26–50% and >50%. The intensity of CD44 positive immunostaining on the cells was graded and classified into four categories (0, 1, 2 and 3) representing negative (0), low (1), moderate (2) and high (3) expression, respectively (Figure 1A–D). For each section, an assessment was made both of staining intensity and of the percentage of cell staining in separate scales. Then, for each section, two scores (intensity and percentage) were added to obtain a score 0 when ≤5% cells stained regardless of the intensity, a score 1 when ≥5% and ≤50% cells stained regardless of the intensity of staining, a score 2 when ≥50% of cells stained with low or moderate intensity, and a score 3 when ≥50% of cells stained with high intensity [15]. 

For statistical analysis, tumors with a final staining score of 0, 1, and 2 were lumped to a low expression group and compared to tumors with a score of 3 as the high expression group.

### 2.3. TCGA Data and Statistical Analysis

The TCGA (https://cancergenome.nih.gov/, accessed on 8 April 2024) publicly available datasets from clear cell RCC patients were downloaded, analyzed, and visualized through the cBioPortal (https://www.cbioportal.org/, accessed on 16 December 2023) [16,17]. RNA sequencing data, Copy Number Variation (CNV) data and methylation data (Infinium HM450 beadchip, Illumina, San Diego, CA, USA) were analyzed and visualized through the cBioPortal. All data generated from immunohistochemical stain scoring, combined with clinicopathological characteristics, were analysed with the SPSS program (SPSS^®^ release 15.0, Chicago, IL, USA). Any *p* value less than 0.05 was considered significant.

## 3. Results

### 3.1. CD44 Protein Expression in Clear Cell RCC and Correlation with Clinicopathological Features

Immunohistochemical staining for CD44 was performed on 89 tumors and the uninvolved adjacent normal tissue. Patient clinicopathological characteristics are presented in Figure 1E. A positive membranous staining reaction for CD44 was observed in 55 (61.8%) patients, while 34 (38.2%) of them were negative. CD44 was found mainly on the tumor cell membrane. Occasionally, both membranous and cytoplasmic staining of weak intensity was observed in cancerous cells. In normal tissue (Figure 1A–D), the intensity of CD44 staining was weakly positive or negative.

CD44 expression had a statistically significant association with TNM stage and more specifically, the CD44-high group of tumors when compared to CD44-low had a higher T stage (*p* < 0.05). Furthermore, the CD44-high group was associated with a higher nuclear grade (*p* < 0.05) in comparison to the CD44-low group of ccRCC tumors (Figure 1F).

### 3.2. CD44 mRNA Expression Inversely Correlates with Patient’s Outcome and Depends on Gene Methylation

**Figure 2:**
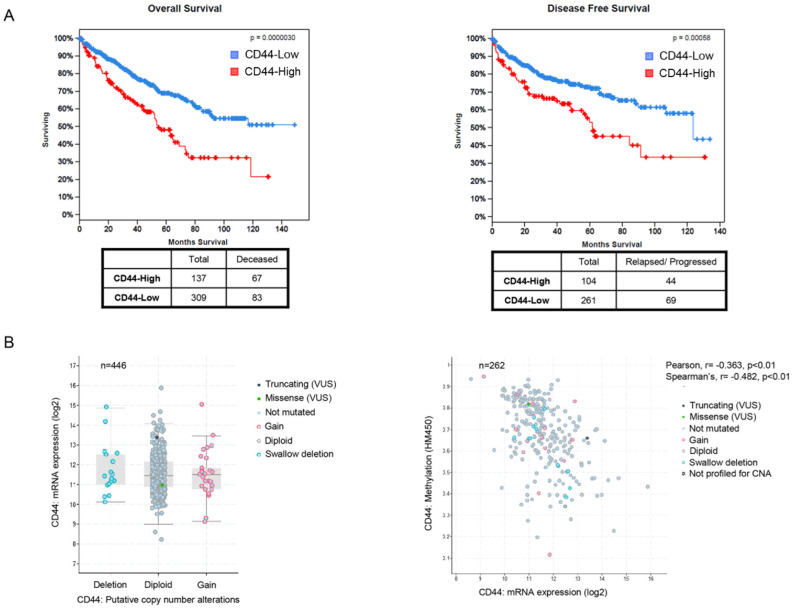
High mRNA expression of CD44 (RNA Seq V2 RSEM data) presents a statistically significant inverse correlation with overall survival (*p* = 0.000003) and disease-free survival (*p* = 0.00058) ((**A**), Left and right panel, respectively). *CD44* mRNA expression (log2 RNA Seq V2 RSEM data) for 446 patients with available data seems to be unaffected by copy number variation (CNV) status as analyzed by the GISTIC algorithm and presented though the cBioPortal ((**B**), Left panel). At the same time, methylation status of the *CD44* gene locus as captured by the HM450 Illumina Beadchip and presented through the cBioPortal, has a statistically significant (Pearson, *p* < 0.01; Spearmann’s, *p* < 0.01) inverse correlation with the *CD44* mRNA expression ((**B**), Left panel).

The above data imply a correlation between high CD44 expression in ccRCC and worse patient outcome. To further explore this association, we analyzed publicly available mRNA (RNA Seq V2 RSEM) expression data from 446 ccRCC patients included in the TCGA database (TCGA, Kidney Renal Clear Cell Carcinoma). A total of 446 and 365 patients (ccRCC) were analyzed for overall survival and disease-free survival, respectively, in conjunction to *CD44* gene expression. Those with high (CD44-High) levels of *CD44* mRNA expression (31%) (z-score threshold used as the cut-off for low and high expression was 0.0) had significantly shorter overall survival (*p* < 0.001) and disease-free survival (*p* < 0.001) than the patients with low levels of *CD44* (Figure 2A, left and right panel, respectively).

In an effort to identify the molecular background of *CD44* gene upregulation in ccRCC, we employed CD44 copy number and methylation data from TCGA, then analyzed and visualized through the cBioPortal (Kidney Renal Clear Cell Carcinoma, https://cbioportal.org/study/summary?id=kirc_tcga_pub, accessed on 16 December 2023) [16,17]. These two mechanisms, copy number variations, and methylation comprise major regulators of gene and mRNA expression and the relevant data are publicly available. Putative copy number alterations analyzed by GISTIC (Genomic Identification of Significant Targets in Cancer) through cBioPortal indicate that the increase in copy number of the *CD44* gene could not explain the observed mRNA upregulation (Figure 2B, left panel). On the other hand, methylation data from the Infinium Human Methylation 450 BeadChip (HM450, Illumina) visualized in cBioPortal for the *CD44* gene indicate that methylation levels present an inverse correlation with *CD44* mRNA expression (Figure 2B, right panel), providing preliminary evidence for the regulation of *CD44* expression by gene methylation in ccRCC. 

## 4. Discussion

Renal cell tumors are a heterogeneous group of neoplasms. Clear cell renal carcinoma is the most common renal malignant tumor with relatively low levels of intratumor heterogeneity. The *CD44* gene is situated on the short arm of chromosome 11, encoding a type I transmembrane-bound glycoprotein with multiple roles in cancerous and normal cells [18]. The most common isoform of CD44 has been termed CD44 standard (CD44s), a protein of about 84KDa that was first isolated from hematopoietic cells [19]. To date, it has been found on a variety of normal tissues such as the epidermis, liver, lung, CNS, cervix, among others [18]. 

Following the identification and characterization of *CD44*, Gunthert and colleagues [20] were able to show that the forced expression of a CD44 variant conferred metastatic potential in a non-metastatic cell line. In addition, soon after the identification of a pro-metastatic role for the CD44 variants, it was demonstrated that a monoclonal antibody against CD44 was able to completely inhibit the binding of human melanoma cells to extracellular hyaluronate in vitro and to inhibit metastases of melanoma cells in vivo [21]. Recent studies indicate that CD44s overexpression is related to tumor grade and overall survival in breast cancer [22], while CD44v6 expression correlates with better patient outcomes in bladder cancer [23]. Although the CD44 variants’ expression in RCC types present contradictory results, CD44 standard seems to be a potential prognostic marker. 

Our results indicate that the increased expression of *CD44* mRNA and protein correlate positively with worse clinical outcomes, as this is denoted through the analysis of tumor size, Furhman grade, and overall survival. It is well known that tumors with a high Furhman grade are associated with a higher risk of local invasion and distant metastases. Our study confirms the previously published reports on the prognostic role of CD44 expression in ccRCC, confirming an association of CD44 with clinicopathological parameters [24,25].

Furthermore, our results provide preliminary evidence of a methylation-dependent upregulation of CD44 in clear cell RCC, excluding possible copy number variations (amplifications or gains) as major genetic drivers of *CD44* mRNA upregulation. This finding further supports the role of epigenetic regulation in ccRCC tumor progression and implicates the use of methylation targeting agents in the treatment of clear cell RCC. In addition, the levels of *CD44* gene methylation alone, or in a relevant gene methylation panel from tumor or cell free DNA, could be employed as a prognostic marker in ccRCC, where relevant to tumor aggressiveness [26]. 

An important limitation of this study is that immunohistochemical analysis of CD44 protein does not distinguish between the 20 possible isoforms of CD44 produced through alternative splicing [10]. Isoform-specific identification could be achieved through CD44 isoform-specific antibodies employed in immunohistochemistry or transcript-specific primers for real-time, and reverse transcription PCR that could quantify isoform gene expression at the mRNA level. While the CD44 isoform-specific antibodies for immunohistochemistry could identify cells in situ that express a specific isoform, the employment of transcript-specific primers on bulk tumor RNA could quantify each transcript level but could not distinguish the cell type (tumor or normal) that expressed a specific CD44 isoform.

Finally, while the measurement of the *CD44* methylation levels could provide a novel prognostic marker for ccRCC aggressiveness as indicated in this analysis, the publicly available methylation data employed in this work need to be confirmed experimentally for the *CD44* gene in an independent ccRCC cohort. In any case, the employment of publicly available data sets in this work was conducted with the aim to extend and support the immunohistochemical findings of CD44 upregulation in our ccRCC patient cohort.

## Data Availability

Bioinformatics data employed in this work are publicly available through the TCGA and cBioPortal, MSK.

## References

[1] American Joint Committee of Cancer (2011). AJCC Cancer Staging Manual.

[2] Delahunt B., Cheville J.C., Martignoni G., Humphrey P.A., Magi-Galluzzi C., McKenney J., Egevad L., Algaba F., Moch H., Grignon D.J. (2013). The International Society of Urological Pathology (ISUP) grading system for renal cell carcinoma and other prognostic parameters. Am. J. Surg. Pathol..

[3] Calvo E., Schmidinger M., Heng D.Y., Grünwald V., Escudier B. (2016). Improvement in survival end points of patients with metastatic renal cell carcinoma through sequential targeted therapy. Cancer Treat. Rev..

[4] Volpe A., Patard J.J. (2010). Prognostic factors of renal cell carcinoma. World J. Urol..

[5] Zisman A., Pantuck AJDorey F., Said J.W., Shvarts O., Quintana D., Gitlitz B.J., Dekernion J.B., Figlin R.A., Belldegrun A.S. (2001). Improved prognostication of renal cell carcinoma using an integrated staging system. J. Clin. Oncol..

[6] Klatte T., Seligson D.B., Leppert J.T., Riggs S.B., Yu H., Zomorodian N., Kabbinavar F.F., Strieter R.M., Belldegrun A.S., Pantuck A.J. (2008). The chemokine receptor CXCR3 is an independent prognostic factor in patients with localized clear cell renal cell carcinoma. J. Urol..

[7] Lam J.S., Klatte T., Kim H.L., Patard J.J., Breda A., Zisman A., Pantuck A.J., Figlin R.A. (2008). Prognostic factors and selection for clinical studies of patients with kidney cancer. Crit. Rev. Oncol. Hematol..

[8] Marhaba R., Zoller M. (2004). CD44 in cancer progression, adhesion, migration and growth regulation. J. Mol. Histol..

[9] Naor D., Sionov R.V., Ish-Shalom D. (1997). CD44 structure function and association with the malignant process. Adv. Cancer Res..

[10] Brown R.L., Reinke L.M., Damerow M.S., Perez D., Chodosh L.A., Yang J., Cheng C. (2011). CD44 splice isoform switching in human and mouse epithelium is essential for epithelial-mesenchymal transition and breast cancer progression. J. Clin. Investig..

[11] Liao H.X., Lee D.M., Levesque M.C., Haynes B.F. (1995). N-terminal and central regions of the human CD44 extracellular domain participate in cell surface hyaluronan binding. J. Immunol..

[12] Zavrides H.N., Zizi-Sermpetzoglou A., Panousopoulos D., Athanasas G., Elemenoglou I., Peros G. (2005). Prognostic evaluation of CD44 expression in correlation with bcl2 and p53 in colorectal cancer. Folia Histochem. Cytobiol..

[13] Bourguignon L.Y., Earle C., Wong G., Spevak C.C., Krueger K. (2012). Stem cell marker (Nanog) and stat-3 signaling promote MicroRNA-2, expression and chemoresistance in hyaluronan/CD44-activated head and neck squamous cell carcinoma cells. Oncogene.

[14] Chen J., Zhou J., Lu J., Xiong H., Shi X., Gong L. (2014). Significance of CD44 expression in head and neck cancer: A systemic review and meta-analysis. BMC Cancer.

[15] Papatheodorou H., Papanastasiou A.D., Sirinian C., Scopa C., Kalofonos H.P., Leotsinidis M., Papadaki H. (2014). Expression patterns of SDF1/CXCR4 in human invasive breast carcinoma and adjacent normal stroma: Correlation with tumor clinicopathological parameters and patient survival. Pathol. Res. Pract..

[16] Cerami E., Gao J., Dogrusoz U., Gross B.E., Sumer S.O., Aksoy B.A., Jacobsen A., Byrne C.J., Heuer M.L., Larsson E. (2012). The cBio Cancer Genomics Portal: An Open Platform for Exploring Multidimensional Cancer Genomics Data. Cancer Discov..

[17] Gao J., Aksoy B.A., Dogrusoz U., Dresdner G., Gross B., Sumer S.O., Sun Y., Jacobsen A., Sinha R., Larsson E. (2013). Integrative analysis of complex cancer genomics and clinical profiles using the cBioPortal. Sci. Signal.

[18] Cichy J., Puré E. (2003). The liberation of CD44. J. Cell Biol..

[19] Screaton G.R., Bell M.V., Jackson D.G., Cornelis F.B., Gerth U., Bell J.I. (1992). Genomic structure of DNA encoding the lymphocyte homing receptor CD44 reveals at least 12 alternatively spliced exons. Proc. Natl. Acad. Sci. USA.

[20] Günthert U., Hofmann M., Rudy W., Reber S., Zöller M., Haussmann I., Matzku S., Wenzel A., Ponta H., Herrlich P. (1991). A new variant of glycoprotein CD44 confers metastatic potential to rat carcinoma cells. Cell.

[21] Guo Y., Ma J., Wang J., Che X., Narula J., Bigby M., Wu M., Sy M.S. (1994). Inhibition of human melanoma growth and metastasis in vivo by anti-CD44 monoclonal antibody. Cancer Res..

[22] Qiao G.L., Song L.N., Deng Z.F., Chen Y., Ma L.J. (2018). Prognostic value of CD44v6 expression in breast cancer: A meta-analysis. OncoTargets Ther..

[23] Klatte T., Seligson D.B., Rao J.Y., Yu H., de Martino M., Garraway I., Wong S.G., Belldegrun A.S., Pantuck A.J. (2010). Absent CD44v6 expression is an independent predictor of poor urothelial bladder cancer outcome. J. Urol..

[24] Lucin K., Matusan K., Dordević G., Stipić D. (2004). Prognostic significance of CD44 molecule in renal cell carcinoma. Croat. Med. J..

[25] Zanjani L.S., Madjd Z., Abolhasani M., Rasti A., Fodstad O., Andersson Y., Asgari M. (2018). Increased expression of CD44 is associated with more aggressive behavior in clear cell renal cell carcinoma. Biomark. Med..

[26] Grammatikaki S., Katifelis H., Farooqi A.A., Stravodimos K., Karamouzis M.V., Souliotis K., Varvaras D., Gazouli M. (2023). An Overview of Epigenetics in Clear Cell Renal Cell Carcinoma. In Vivo.

